# Increased transgene expression level of rabies virus vector for transsynaptic tracing

**DOI:** 10.1371/journal.pone.0180960

**Published:** 2017-07-10

**Authors:** Shinya Ohara, Yasuhiro Sota, Sho Sato, Ken-Ichiro Tsutsui, Toshio Iijima

**Affiliations:** Division of Systems Neuroscience, Tohoku University Graduate School of Life Sciences, Sendai, Japan; French National Centre for Scientific Research, FRANCE

## Abstract

Viral vectors that can infect neurons transsynaptically and can strongly express foreign genes are useful for investigating the organization of neural circuits. We previously developed a propagation-competent rabies virus (RV) vector based on a highly attenuated HEP-Flury strain (rHEP5.0-CVSG), which selectively infects neurons and propagates between synaptically connected neurons in a retrograde direction. Its relatively low level of transgene expression, however, makes immunostaining necessary to visualize the morphological features of infected neurons. To increase the transgene expression level of this RV vector, in this study we focused on two viral proteins: the large protein (L) and matrix protein (M). We first attempted to enhance the expression of L, which is a viral RNA polymerase, by deleting the extra transcription unit and shortening the intergenic region between the G and L genes. This viral vector (rHEP5.0-GctL) showed increased transgene expression level with efficient transsynaptic transport. We next constructed an RV vector with a rearranged gene order (rHEP5.0-GML) with the aim to suppress the expression of M, which plays a regulatory role in virus RNA synthesis. Although this vector showed high transgene expression level, the efficiency of transsynaptic transport was low. To further evaluate the usability of rHEP5.0-GctL as a transsynaptic tracer, we inserted a fluorescent timer as a transgene, which changes the color of its fluorescence from blue to red over time. This viral vector enabled us the differentiation of primary infected neurons from secondary infected neurons in terms of the fluorescence wavelength. We expect this propagation-competent RV vector to be useful for elucidating the complex organization of the central nervous system.

## Introduction

Transsynaptic tracers are useful tools to reveal the hierarchical connectivity in the central nervous system. Neurotropic viruses that can propagate within synaptically connected neural circuits and amplify signals through replication, such as the herpes simplex virus type 1, the pseudorabies virus, and the rabies virus (RV), have been used as such an anatomical tool [1–3]. Among these viruses, RV is preferred owing to its ability to selectively infect neurons and low cytotoxicity [4–6].

We previously developed a recombinant RV vector based on a vaccinated HEP-Flury strain (HEP) [7], since vaccinated strains show higher levels of transcription than pathogenic strains [8,9]. This vector was further developed by replacing the glycoprotein (G) gene of HEP with that of CVS, and adding an additional transgene insertion site between the N and P genes [10]. Since this recombinant RV vector (rHEP5.0-CVSG) efficiently propagated transsynaptically in a retrograde direction and expressed transgene in the infected neuron, this viral vector could be used as a potential tool for selective gene delivery in the central nervous system. Note that this RV vector differs from G-gene deleted RV vectors [11–15], which are now widely used in the field of neuroscience, in terms that this vector can propagate transsynaptically without supplying the G-gene *in trans* within the infected cells. By using this propagation-competent RV vector, we have revealed the mutlisynaptic connections in the medial temporal lobe memory system [16,17].

Although our RV vector was designed to express the transgene at high levels, the transgene expression level of this propagation-competent RV vector was significantly lower than that of the G-deleted RV vector, and expressed marker proteins must be immunostained to clearly visualize the morphological features of infected neurons [11]. Increasing the transgene expression level will expand the usability of this vector as a neurotracing tool since it will not only enable us to examine the morphology of targeted neurons without requiring any staining procedure, but may solve one of the pitfalls of transsynaptic tracing. In poly(trans)synaptic tracing, several samples with different survival times must be prepared to distinguish primary infected neurons (1^st^-order neurons) from secondary infected ones (2^nd^-order neurons). This requires the use of many experimental animals with accurate injection. This problem may be solved by using a propagation-competent RV vector with a high expression level of a fluorescent timer, which changes the color of its fluorescence over time [18], since it would enable the differentiation between the 1^st^- and the 2^nd^-order neurons in the same sample.

To develop a recombinant RV vector with a high level of transgene expression, in this study we focused on two viral proteins: the large protein (L) and the matrix protein (M). The L gene encodes the viral RNA polymerase, and it has been reported that the overexpressed L gene increases viral-gene mRNA transcripts and the expression level of nucleoprotein (N) and phosphoprotein (P) [19,20]. Indeed, we have recently shown that the enhanced transgene expression of a G-deleted RV vector compared with a propagation-competent RV vector is partially due to the increased transcription level of the L gene [11,21]. M protein is mainly responsible for the assembly and budding of bullet-shaped viral particles [22], but is also a regulatory element that balances viral transcription and replication [8,20,23,24]. It has been reported that a recombinant RV with an attenuated M expression shows a high-transcription-level phenotype [24].

We constructed an RV vector with deleted extra transcription unit and modified intergenic region between the G and L genes with the aim to enhance the expression of the L gene (rHEP5.0-GctL). To construct an RV vector with attenuated M-gene expression (rHEP5.0-GML), we modified the order of the M and G genes. As we expected, rHEP5.0-GctL and rHEP5.0-GML showed higher transgene expression levels than rHEP5.0-CVSG *in vitro* and *in vivo*. rHEP5.0-GctL, which shows a high transgene expression level and efficient transsynaptic transport, enabled not only the visualization of infected neurons without requiring any staining but also the differentiation of secondary infected neurons from primary infected neurons in terms of the fluorescence wavelength of the expressed fluorescent timer.

## Materials and methods

### Plasmid construction and virus recovery

To construct an RV vector with enhanced L gene expression (rHEP5.0-GctL), the viral transcriptional unit between the G and L genes was deleted from pHEP5.0-CVSG (DDBJ/GenBank/EMBL accession number, AB839170). A PCR fragment spanning the 5’-noncoding region upstream of the L gene and the 5’-terminal part of the L gene was amplified using the primers L-BsiWI-5 (5’- AACCGTACGGAGACCCATATCAAGATGCTGGATCCGGGAG-3’; the *Bsi* WI site is underlined) and L-ClaI-3 (5’- ACAAGATCGATCTGTTGCCTTCTTTCATAGTGGTTG-3’; the *Cla* I site is underlined). The sequence between the *Bsi* WI site and the *Cla* I site was removed from pHEP5.0-CVSG and replaced with the PCR fragment shown above. As a result, in addition to the deletion of transcription unit between G and L genes, the IGR upstream of the L gene was shortened from 24 nucleotides to 2 nucleotides. The resulting plasmid was designated as pHEP5.0-GctL.

To construct an RV vector with attenuated M-gene expression (rHEP5.0-GML), the positions of the M gene and G gene of pHEP5.0-CVSG were exchanged, since it has been reported that viral genes located closer to the 3’ end show higher expression levels than downstream genes [25–27]. We first constructed an M-gene-deleted RV vector (pHEP5.0-CVSG-ΔM) by inserting the following two PCR fragments into pHEP5.0-CVSG. The fragment containing the *Blp* I site, the 3’-terminal part of the P gene, and the *Nhe* I site was amplified using the primers P-BlpI-5 (5’-CAAGCTAAGCAAAATCATGCAAGATGA-3’; the *Blp* I site is underlined) and P-NheI-3 (5’-GTTGCTAGCTTTTTTTCATATCGACTCC-3’; the *Nhe* I site is underlined). The fragment containing the *Spe* I site, CVS-G gene, and *Bsi* WI site was amplified using the primers G-SpeI-5 (5’-AAAACTAGTAACATCCCTCAAAAGACTTAAGGA-3’; the *Spe* I site is underlined) and G-BsiWI-3 (5’-AATCGTACGAGAGGTGT-3’; the *Bsi* WI site is underlined). The two PCR fragments were ligated utilizing the *Nhe* I site and *Spe* I site, which produce compatible cohesive ends, and inserted between the *Blp* I site and *Bsi* WI site of pHEP5.0-CVSG. The length of IGR at the border between the P and G genes was 6 nt in the resulting plasmid pHEP5.0-CVSG-ΔM. In the next step, we amplified a PCR fragment of the M gene containing the *Bsi* WI site and *Nhe* I site using the primers M-BsiWI-5 (5’- ACCCGTACGAAAATGAACTTTCTATGT-3’; the *Bsi* WI site is underlined) and M-NheI-3 (5’-ATTGCTAGCTTATTCTAAAAGCAGAGAAGAGTCTTTG-3’; the *Nhe* I site is underlined). We then inserted this fragment into the transgene insertion site between the G and L genes of pHEP5.0-CVSG-ΔM using the *Bsi* WI and *Nhe* I restriction sites. The resulting plasmid was designated as pHEP5.0-GML.

**Fig 1:**
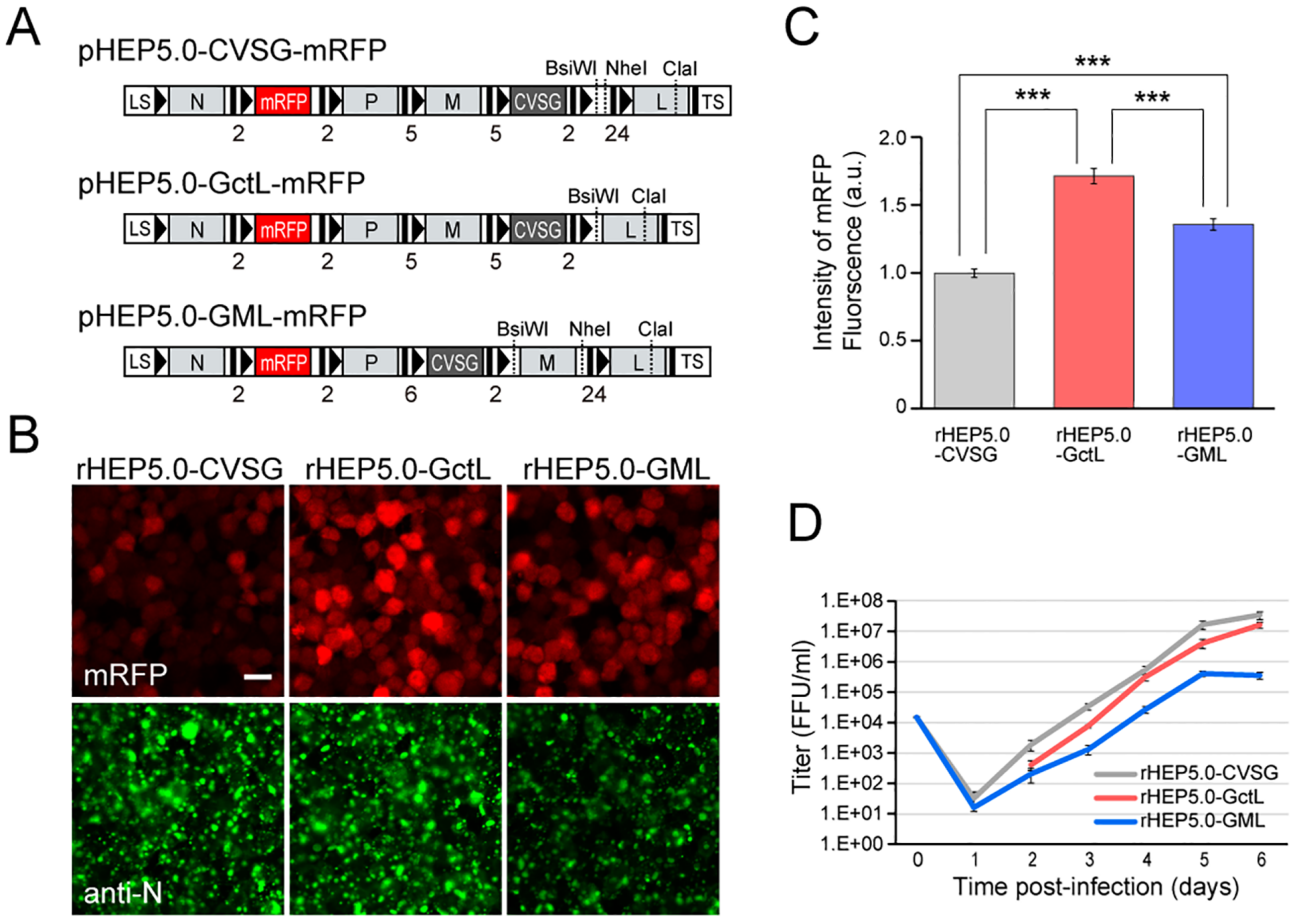
Characteristics of RV vectors in cultured cells. **A**: Genome organization of recombinant RV vectors. The transcription start and stop/polyadenylation signals are respectively indicated by black bars and black arrowheads in the schematic diagram, and the number of nucleotides of IGR are shown below the diagram. LS, leader sequence; TS, trailer sequence. **B**: Photomicrographs of RV-infected NA cells at 2 dpi. The three RV vectors expressed the transgene mRFP (red), which was inserted between the N and P genes. Infection of the viral vector can be confirmed by immunofluorescence of the N protein (green). Scale bar = 20 μm. **C**: Fluorescence intensities of mRFP in infected cells at 2 dpi [mean ± standard errors, numbers of analyzed cells: 189 (rHEP5.0-CVSG), 382 (rHEP5.0-GctL), 276 (rHEP5.0-GML), *** *p* < 0.001]. **D**: Viral titer growth curves of RV vectors (mean ± standard errors, N = 6). No viruses were detected in rHEP-5.0-GctL-infected cells at 1 dpi.

In this study, we used RV vectors that express mRFP as a transgene [28]. A PCR fragment containing the *Not* I site, mRFP open reading frame, and *Sac* II site was inserted into the additional transcription insertion unit between the N and P genes of pHEP5.0-CVSG, pHEP5.0-GctL, pHEP5.0-GML. The resulting plasmids were designated pHEP5.0-CVSG-mRFP, pHEP5.0-GctL-mRFP, and pHEP5.0-GML-mRFP, respectively (Fig 1A).

To construct a recombinant RV that highly expresses a slow fluorescent timer (sFT) [18], one to three copies of sFT were inserted into pHEP5.0-GctL as transgenes. An RV vector with a single copy of sFT (pHEP5.0-GctL-sFT) was constructed by inserting a PCR fragment containing the *Not* I site, sFT open reading frame, and *Sac* II site into the additional transcription insertion unit between the N and P genes of pHEP5.0-GctL. An RV vector with two copies of sFT (pHEP5.0-GctL-FPF) was constructed by inserting two genes of sFT linked by a P2A self-cleavage sequence (FPF). Two PCR fragments containing the sFT sequence were inserted in the synthesized sequence (5’-GAATTCAAACGTACG**GGAAGCGGAGCTACTAACTTCAGCCTGCTGAAGCAGGCTGGAGACGTGGAGGAGAACCCTGGACCT**TCCGGAAAAGCGGCCGC -3’; the *Eco* RI, *Bs*i WI, *Bsp* EI, and *Not* I sites are underlined in this order; the P2A sequence is shown in bold) using the following primers. sFT-EcoRI-NotI-5 (5’- ATTGAATTC GCGGCCGCACCATGGTGAGCAAGGGCGAGGAGGATAA-3’; the *Eco* RI and *Not* I sites are underlined in order) and sFT-BsiWI-3 (5’- GTTCGTACGCTTGTACAGCTCGTCCATGCCGCCGGTGGAGTG-3’; the *Bsi* WI is underlined) were used as primers for the first sFT fragment, and sFT-BspEI-5 (5’-ATTTCCGGAACCATGGTGAGCAAGGGCGAGGAGGATAA-3’; the *Bsp* EI site is underlined) and sFT-NotI-SacII-3 (5’- GTTGCGGCCGCGGTTACTTGTACAGCTCGTCCATGCC -3’; the *Not* I and *Sac* II sites are underlined) for the second sFT fragment. The FPF sequence was inserted into pHEP5.0-GctL using *Not* I and *Sac* II. An RV vector with three copies of sFT (pHEP5.0-GctL-FTFPF) was constructed by inserting three genes of sFT linked by the T2A- and P2A-self-cleavage sequence (FTFPF). Three PCR fragments containing the sFT sequence were inserted into the synthesized sequence (5’- GAATTCAAACCCGGGAAATCTAGA**GGAAGCGGAGAGGGCAGAGGAAGTCTGCTAACATGCGGTGACGTCGAGGAGAATCCTGGACCT**GCATGCAAACGTACG**GGAAGCGGAGCTACTAACTTCAGCCTGCTGAAGCAGGCTGGAGACGTGGAGGAGAACCCTGGACCT**TCCGGAAAAGCGGCCGC -3’; the *Eco* RI, *Xma* I, *Xba* I, *Sph* I, *Bsi* WI, *Bsp* EI, and *Not* I sites are underlined in this order; the T2A and P2A sequence is shown in bold) using the following primers. sFT-EcoRI-NotI-5 and sFT-XbaI-3 (5’- GTTTCTAGACTTGTACAGCTCGTCCATGCCGCCGGTGGAGTG-3’; the Xba I site is underlined) were used as primers for the first sFT fragment, sFT-SphI-5 (5’- ATTGCATGCACCATGGTGAGCAAGGGCGAGGAGGATAA-3’; the Sph I site is underlined) and sFT-BsiWI-3 for the second sFT fragment, and sFT-BspEI-5 and sFT-NotI-SacII-3 for the third sFT fragment. The FTFPF sequence was inserted into pHEP5.0-GctL using *Not* I and *Sac* II.

The recombinant RV vectors were recovered using mouse neuroblastoma cells of A/J mouse origin (NA) as described previously [7]. The recovered viruses generated from pHEP5.0-CVSG-mRFP, pHEP5.0-GctL-mRFP, pHEP5.0-GML-mRFP, pHEP5.0-GctL-sFT, pHEP5.0-GctL-FPF, and pHEP5.0-GctL-FTFPF were designated rHEP5.0-CVSG-mRFP, rHEP5.0-GctL-mRFP, rHEP5.0-GML-mRFP, rHEP5.0-GctL-sFT, rHEP5.0-GctL-FPF, and rHEP5.0-GctL-FTFPF, respectively. All viral strains were filtered and concentrated by ultracentrifugation. A viral suspension was kept in small aliquots at −80°C. Each aliquot was thawed in a safety cabinet before each experiment. To determine the viral titer, we conducted a direct florescence test using NA cells as described elsewhere [29].

### Viral infection in cultured cells

NA cells were plated on glass coverslips in a 4-well plate and maintained at 37°C in minimum essential medium supplemented with 10% heat-inactivated fetal bovine serum (NA culture medium). To evaluate the efficiency of mRFP expression and the cytotoxicity of the mRFP-expressing RV vectors, each RV vector was applied to the dish at a multiplicity of infection (MOI) of 10. Six hours after infection the inoculum was replaced with a fresh medium and cells were incubated at 34°C. The fluorescence intensity of mRFP was examined two days and six days postinfection (dpi). Cells were fixed for 1 h at 4°C in phosphate-buffered saline (PBS) containing 4% paraformaldehyde, washed with PBS three times, and then soaked for 1 h at room temperature in PBS containing 5% goat serum and 0.1% Triton X-100. Cells were then incubated overnight at 4°C with monospecific rabbit anti-N antiserum [30] diluted in PBS containing 0.1% Triton X-100 and 0.025% NaN_3_. After the primary antibody was aspirated, cells were washed and permeabilized in PBS containing 0.1% Triton X-100 (PBT). Cells were then incubated for 4 h at room temperature in Alexa Fluor 488-conjugated anti-rabbit goat IgG (1:300; Jackson ImmunoResearch) and Hoechst 33258 solution (1:1000; Dojindo) diluted in PBT, and were washed 3 times with PBS. Fluorescence labeling was assessed using a confocal laser-scanning microscope (LSM 5 Exciter, Carl Zeiss). The fluorescence intensity of mRFP was quantified by measuring the average fluorescence intensity per pixel of the infected neurons with ImageJ software (http://rsb.info.nih.gov/ij) and normalized on the basis of the fluorescence intensity of rHEP5.0-CVSG-mRFP-infected cells. The apoptotic cells were assessed by examining nuclear DNA fragmentation by the terminal deoxynucleotidyl transferase dUTP nick-end labeling (TUNEL) method (In situ apoptosis detection kit, Takara). To prevent detachment of the cells, Poly-L-Lysine coated dishes were used for this apoptosis detection.

To assess the multistep-growth curves of the RV vectors, confluent NA cell monolayers grown in 6-well plates were infected with the vectors at MOI of 0.01. After 1 h incubation at 34°C, the inoculum was washed twice with NA culture medium and fresh medium was added. The inoculum was further incubated at 34°C. Supernatant was harvested at the indicated time points, and the virus was titrated using NA cells. Differences in viral titer at each time point were analyzed by two-way ANOVA with Bonferroni’s multiple comparison test.

To evaluate the sFT expression levels of rHEP5.0-GctL-sFT, rHEP5.0-GctL-FPF, and rHEP5.0-GctL-FTFPF, each virus was applied to NA cells at MOI of 20. The blue form of sFT was imaged using the confocal laser-scanning microscope LSM 5 Exciter after two days of infection. Fluorescence intensity was quantified by measuring the average fluorescence intensity per pixel with ImageJ software and normalized on the basis of rHEP5.0- GctL-sFT-infected cells.

### Viral infection *in vivo*

Young adult male Wistar rats weighing 200–230 g were used in this study. All experiments were approved by the Center for Laboratory Animal Research, Tohoku University, and conducted in accordance with the Guidelines of the National Institutes of Health and the Guidelines for Animal Care and Use published by our institute. We set clinical signs of rabies (slow and circular movements, paralysis, cachexia) as humane endpoints. However, since none of the rats showed any clinical signs of rabies, they were all sacrificed with an overdose of sodium pentobarbital after a certain survival time in accordance with the experimental schedule. All experiments requiring injections of recombinant RV vectors were carried out in a special laboratory (biosafety level 2) designed for *in vivo* infectious experiments.

Rats were deeply anaesthetized with ketamine (60 mg/kg, i.p.) and xylazine (4.8 mg/kg, i.p.) and mounted on a stereotaxic frame. The skull was exposed, and a small burr hole was drilled above the injection site. Injections were carried out using a glass micropipette (tip diameter = 30 μm) connected to a 2-μl Hamilton microsyringe. To evaluate mRFP expression and transsynaptic transport *in vivo*, either 600 nl of rHEP5.0-CVSG-mRFP (3.0×10^8^ FFU/ml, n = 3 for 3 dpi, n = 4 for 5 dpi), rHEP5.0-GctL-mRFP (3.0×10^8^ FFU/ml, n = 2 for 3 dpi, n = 2 for 5 dpi), or rHEP5.0-GML-mRFP (3.0×10^8^ FFU/ml, n = 2 for 3 dpi, n = 3 for 5 dpi) was injected into the medial entorhinal cortex (MEC). Each virus was injected with 1% pontamine sky blue so that the injection sites could be located. After the injection at 60 nl/min, the pipette was left in place for another 10 min before it was withdrawn. The skin wound was sutured, and the animal was monitored for recovery from anesthesia and returned to its home cage. Throughout the survival period, the rats were kept inside a small safety cabinet. After a survival period of either 3 or 5 days, the animals were deeply anaesthetized with sodium pentobarbital (100 mg/kg, i.p.) and transcardially perfused and fixed with 10% sucrose in 0.1 M phosphate buffer (PB; pH 7.4) followed by 4% paraformaldehyde in 0.1 M PB. The brains were removed from the skulls, postfixed in the same fresh fixative for 4 h at 4°C and then cryoprotected for at least 48 h at 4°C in PB containing 30% sucrose. The brains were coronally sectioned at 50 μm on a freezing microtome. An immunostaining procedure similar to that used for the *in vitro* study was conducted to visualize RV-infected neurons. To clearly visualize the infected neurons and count them in CA3, the sections were also stained with with rabbit anti-RFP antibody (1:400; Molecular Probes). The distribution of labeled neurons was examined under a Zeiss Axiovert 200M and imaged using a digital camera (AxioCam MRm). To quantify mRFP fluorescence intensity, the images of infected cells were captured using a laser scanning confocal microscope (LSM 5 Exciter, Carl Zeiss) under identical conditions between different viral vectors. The average intensity per pixel of mRFP fluorescence in the soma was measured using ImageJ software and normalized on the basis of the fluorescence intensity of rHEP5.0-CVSG-mRFP-infected neurons. Differences in fluorescence intensity and the number of infected CA3 cells were analyzed by two-way ANOVA with Bonferroni’s multiple comparison test.

To evaluate sFT labeling, 1800 nl of rHEP5.0-GctL-FTFPF (2.2×10^9^ FFU/ml, n = 4) was injected into MEC, and the animal was perfused and fixed after six days of survival. The brain was sectioned at 60 μm and the sFT native fluorescence of both the blue and red forms was imaged using the confocal laser-scanning microscope LSM 5 Exciter. The average fluorescence intensity per pixel was measured using ImageJ software, and the red-to-blue ratio was calculated. The fluorescence intensity ratio of CA3-infected cells was normalized on the basis of that of CA1-infected cells. The statistical definition for identifying a Blue- or Red-neuron was determined by classifying the fluorescence ratio of each infected neuron into one of two clusters by k-means clustering.

## Results

### Effects of intergenic region, transcription unit, and gene order modifications on transgene expression

We first attempted to increase the expression level of the L gene, which is related to viral transcription and replication. We previously have shown that the deletion of the upstream viral gene with its transcription unit results in an increase of L gene transcription [21]. In addition, it has been reported that the expression level of the L gene changes depending on the length of the sequence of IGR between the G gene and the L gene, and that a shorter IGR in front of the L gene enhances viral gene expression via enhanced L expression [19]. Thus, to increase the expression level of the L gene, we deleted the extra transcription unit between the G and L genes, and shortened the IGR upstream of the L gene from 24 nucleotides to 2 nucleotides (rHEP5.0-GctL, Fig 1A). We also focused on the M gene, which has been suggested to regulate the balance between virus transcription and replication [24]. To attenuate the expression of M, we shifted the position of the M gene, which was located upstream of the G gene, to downstream of the G gene (rHEP5.0-GML, Fig 1A).

The mRFP gene was inserted into the transgene insertion site between the N and P genes (Fig 1A), and the mRFP expression levels of rHEP5.0-GctL-mRFP and rHEP5.0-GML-mRFP were compared with that of rHEP5.0-CVSG-mRFP in NA cells. Cells were infected with one of these three viruses, and the fluorescence intensity of mRFP was examined at 2 dpi (Fig 1B and 1C) and 6 dpi (S1 Fig). At 2 dpi the mean fluorescence intensities of the rHEP5.0-GctL-mRFP- and rHEP5.0-GML-mRFP-infected cells were significantly higher than that of the rHEP5.0-CVSG-mRFP-infected cells, and the rHEP5.0-GctL-mRFP-infected cells showed a significantly higher fluorescence intensity than the rHEP5.0-GML-mRFP-infected cells (*p* < 0.0001, one-way ANOVA; post-hoc t-test, Bonferroni-corrected, *p* < 0.00033 for all pairs, Fig 1B and 1C). Six days after infection, the degree of intensity difference increased between rHEP5.0-CVSG-mRFP and the other two vectors (*p* < 0.0001, one-way ANOVA; post-hoc t-test, Bonferroni-corrected, *p* < 0.00033 for rHEP5.0-CVSG-mRFP infection vs rHEP5.0-GctL-mRFP infection and rHEP5.0-CVSG-mRFP infection vs rHEP5.0-GML-mRFP infection, S1A and S1B Fig). This result indicates that the transgene expression levels of rHEP5.0-GctL and rHEP5.0-GML are higher than that of rHEP5.0-CVSG. There were also differences in the intensity of anti-N antibody labeling between these three vectors at 6 dpi (S1C Fig). The intensity of anti-N antibody labeling of rHEP5.0-GctL-mRFP-infected cells was higher than that of rHEP5.0-CVSG-mRFP-infected cells, and the intensity of anti-N antibody labeling of rHEP5.0-GML-mRFP-infected cells was higher than that of rHEP5.0-CVSG-mRFP- and rHEP5.0-GctL-mRFP-infected cells (*p* < 0.0001, one-way ANOVA; post-hoc t-test, Bonferroni-corrected, *p* < 0.0033 for rHEP5.0-CVSG-mRFP infection vs rHEP5.0-GctL-mRFP infection, *p* < 0.00033 for rHEP5.0-CVSG-mRFP infection vs rHEP5.0-GML-mRFP infection, and rHEP5.0-GctL-mRFP infection vs rHEP5.0-GML-mRFP infection).

We also observed differences in the number of attached cells among the three dishes with cells infected with these three viral vectors after long periods (S1A Fig). Although there were no significant differences in the number of attached cells among these three dishes at 2 dpi (*p* = 0.90, one-way ANOVA), the numbers of cells in the rHEP5.0-GctL-mRFP- and rHEP5.0-GML-mRFP-infected dishes became significantly smaller than that in the rHEP5.0-CVSG-mRFP-infected dish at 6 dpi (*p* < 0.0001, one-way ANOVA; post-hoc t-test, Bonferroni-corrected, *p* < 0.0033 for rHEP5.0-CVSG-mRFP infection vs rHEP5.0-GctL-mRFP infection and rHEP5.0-CVSG-mRFP infection vs rHEP5.0-GML-mRFP infection, S1D Fig). To examine the extent of apoptosis induced by these three viruses, we compared the percentage of cells with fragmented nuclei using the TUNEL technique. Although TUNEL-positive cells were hardly observed at 2 dpi in either of the infected dishes, the number of TUNEL-positive cells increased at 5 dpi (S2A Fig). The percentage of TUNEL-positive cells in the rHEP5.0-GctL-mRFP- and rHEP5.0-GML-mRFP-infected dishes were significantly larger than that in the rHEP5.0-CVSG-mRFP-infected dish (*p* < 0.01, one-way ANOVA; post-hoc t-test, Bonferroni-corrected, *p* < 0.016 for rHEP5.0-CVSG-mRFP infection vs rHEP5.0-GctL-mRFP infection and rHEP5.0-CVSG-mRFP infection vs rHEP5.0-GML-mRFP infection, S2B Fig).

The efficiency of viral production is also an important factor for a transsynaptic viral vector since it likely affects the efficiency of viral propagation. To determine whether the modifications affect viral growth kinetics, we compared the mutistep-growth curves of the three viral vectors in NA cells (Fig 1D). rHEP5.0-GML-mRFP showed the lowest growth rate among all viruses (*p* < 0.05 at 2 dpi, one-way ANOVA; post-hoc t-test, Bonferroni-corrected, *p* < 0.016 for rHEP5.0-CVSG-mRFP vs rHEP5.0-GML-mRFP, *p* < 0.0001 at each time point from 3 to 6 dpi, one-way ANOVA; post-hoc t-test, Bonferroni-corrected, *p* < 0.00033 for rHEP5.0-CVSG-mRFP vs rHEP5.0-GML-mRFP at 3–6 dpi and rHEP5.0-CVSG-mRFP vs rHEP5.0-GctL-mRFP at 4–6 dpi, *p* < 0.0033 for rHEP5.0-CVSG-mRFP vs rHEP5.0-GctL-mRFP at 3 dpi), and its titer was 100-fold lower than that of parental rHEP5.0-CVSG-mRFP and 50-fold lower than that of rHEP5.0-GctL-mRFP at 6 dpi. The growth curve of rHEP5.0-CVSG-mRFP and rHEP5.0-GctL-mRFP was comparable, and there was no significant difference in viral titer between these two viruses except at 5 dpi (post-hoc t-test, Bonferroni-corrected, *p* < 0.0033 for rHEP5.0-CVSG-mRFP vs rHEP5.0-GctL-mRFP at 5 dpi).

**Fig 2:**
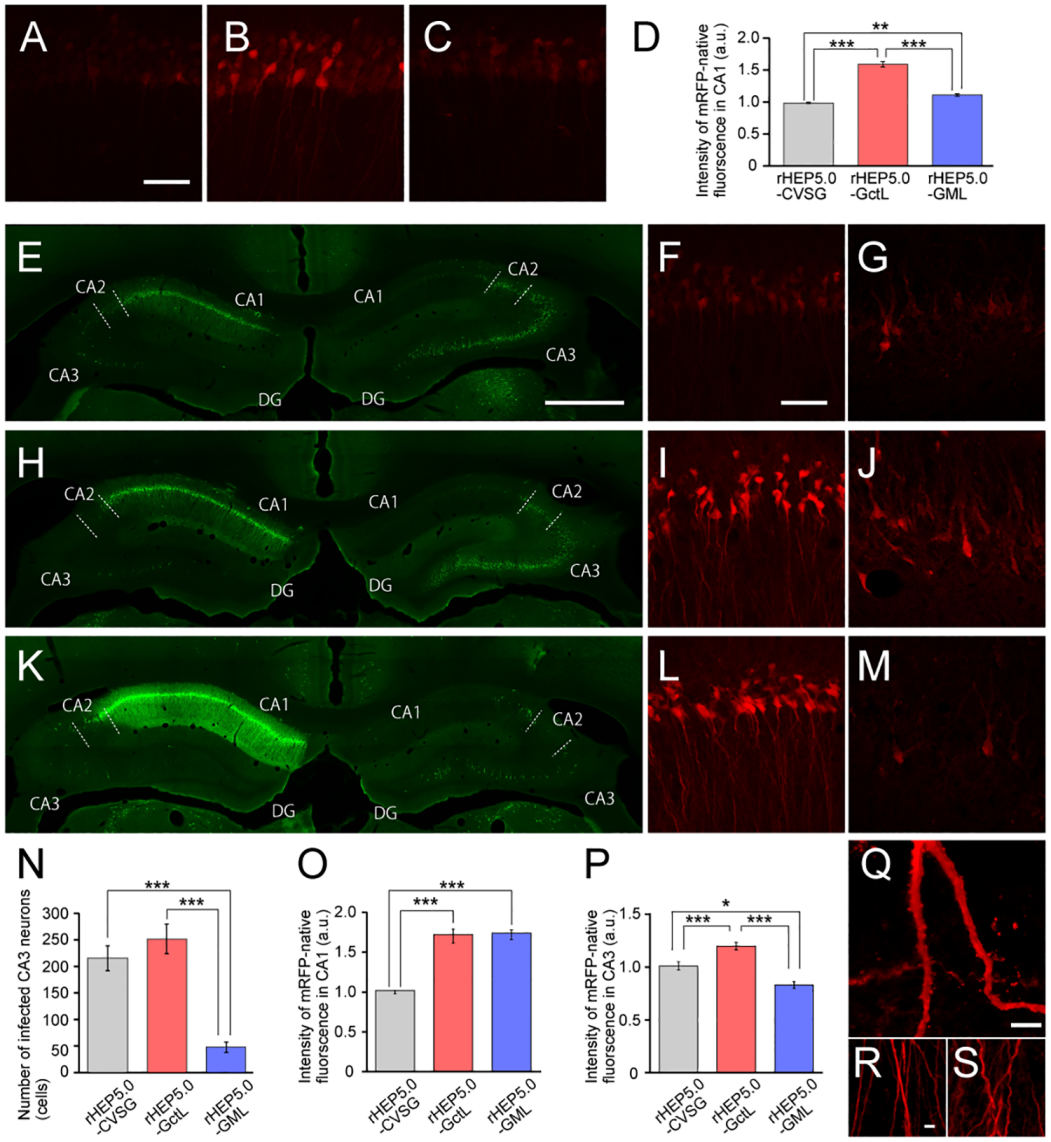
Characteristics of RV vectors *in vivo*. **A-C**: Fluorescence photomicrographs demonstrating mRFP fluorescence of infected CA1 at 3 dpi of either rHEP-CVSG-mRFP (A), rHEP-GctL-mRFP (B), or rHEP-GML-mRFP (C) into MEC of left hemisphere. Scale bar = 50 μm in (A) [applies to (B) and (C)]. **D**: Fluorescence intensity of infected CA1 neurons [mean ± standard errors, number of analyzed cells: 129 (rHEP5.0-CVSG), 160 (rHEP5.0-GctL), and 149 (rHEP5.0-GML), *** *p* < 0.001, * *p* < 0.05]. **E-M**: Fluorescence photomicrographs demonstrating immunoreactivity against N protein in dorsal hippocampus (E, H, K) and mRFP fluorescence of infected CA1 (F, I, L) and contralateral CA3 neurons (G, J, M) at 6 dpi of either rHEP-CVSG-mRFP (E-G), rHEP-GctL-mRFP (H-J), or rHEP-GML-mRFP (K-M) into MEC of left hemisphere. CA3 (G, J, M) was imaged at higher laser power and optical gain than CA1 (F, I, L). Scale bar = 1000 μm in (E) [applies to (H) and (K)], 50 μm in (G) [applies to (G), (I), (J), (L), and (M)]. **N**: Number of infected neurons in CA3 per section [mean ± standard errors, number of analyzed sections: 6 (rHEP5.0-CVSG), 4 (rHEP5.0-GctL), and 6 (rHEP5.0-GML), *** *p* < 0.001]. **O-P**: Fluorescence intensity of infected CA1 (O) and CA3 (P) neurons [mean ± standard errors, number of analyzed cells: 346 (rHEP5.0-CVSG), 225 (rHEP5.0-GctL), and 234 (rHEP5.0-GML) for CA1; 48 (rHEP5.0-CVSG), 50 (rHEP5.0-GctL), and 19 (rHEP5.0-GML) for CA3, *** *p* < 0.001, * *p* < 0.05]. **Q**: High-magnification photomicrographs of rHEP-GctL-mRFP-infected neurons showing labeled dendritic spines. Scale bar = 5 μm. **R-S**: Apical dendrites of CA1 pyramidal neurons infected with rHEP-GctL-mRFP (R) and rHEP-GML-mRFP (R). Scale bar = 5 μm in (R) applies to (S).

We next examined the expression levels of rHEP5.0-GctL-mRFP, rHEP5.0-GML-mRFP, and rHEP5.0-CVSG-mRFP *in vivo* by injecting these viral vectors into the deep layer of MEC of the rat. We first examined the labeling of infected neurons at 3 dpi, which is a suitable survival period to examine the 1^st^-order neurons [16,17]. 1^st^-order neurons were observed in the ipsilateral CA1 region which projects directly to the deep layer of MEC (Fig 2A–2C). Similar to the *in vitro* results at 2 dpi (Fig 1C), the mean mRFP fluorescence intensity of rHEP5.0-GctL-mRFP- and rHEP5.0-GML-mRFP-infected cells were significantly higher than that of the rHEP5.0-CVSG-mRFP-infected cells, and the rHEP5.0-GctL-mRFP-infected cells showed a significantly higher fluorescence intensity than the rHEP5.0-GML-mRFP-infected cells (*p* < 0.0001, one-way ANOVA; post-hoc t-test, Bonferroni-corrected, *p* < 0.00033 for rHEP5.0-CVSG-mRFP infection vs rHEP5.0-GctL-mRFP infection and rHEP5.0-GctL-mRFP infection vs rHEP5.0-GML-mRFP infection, *p* < 0.0033 for rHEP5.0-CVSG-mRFP infection vs rHEP5.0-GML-mRFP infection, Fig 2D). We next examined the labeling of infected neurons at 5 dpi, which is a suitable survival period for examining both the 1^st^- and 2^nd^-order neurons [16,17]. In addition to the 1^st^-order neurons in ipsilateral CA1, 2^nd^-order neurons was observed in the ipsi- and contralateral CA3 regions, which indirectly project to MEC via CA1 region. Numerous infected neurons were observed in both the CA1 and CA3 regions in rats injected with either rHEP5.0-GctL-mRFP or rHEP5.0-CVSG-mRFP (Fig 2E and 2H). In agreement with our previous study [16], the number of labeled neurons was larger in the contralateral CA3 than in the ipsilateral CA3 region in these samples. Similar to the *in vitro* results, the mean mRFP fluorescence intensity of rHEP5.0-GctL-mRFP-infected cells was higher than that of the rHEP5.0-CVSG-mRFP-infected cells both in CA1 (*p* < 0.0001, one-way ANOVA; post-hoc t-test, Bonferroni-corrected, *p* < 0.00033 for rHEP5.0-CVSG-mRFP infection vs rHEP5.0-GctL-mRFP infection, Fig 2F, 2I and 2O) and CA3 (*p* < 0.0001, one-way ANOVA; post-hoc t-test, Bonferroni-corrected, *p* < 0.00033 for rHEP5.0-CVSG-mRFP infection vs rHEP5.0-GctL-mRFP infection, Fig 2G, 2J and 2P). The detailed structure, including the dendritic spines, of the CA1 neurons could be observed with the native mRFP fluorescence expressed by rHEP5.0-GctL-mRFP (Fig 2Q), and no apparent morphological changes were observed in the infected neurons (Fig 2R). In contrast to these two vectors, the number of infected CA3 neurons were significantly lower in the rHEP5.0-GML-mRFP-injected sample (*p* < 0.0001, one-way ANOVA; post-hoc t-test, Bonferroni-corrected, *p* < 0.00033 for rHEP5.0-CVSG-mRFP infection vs rHEP5.0-GML-mRFP and rHEP5.0-GctL-mRFP infection vs rHEP5.0-GML-mRFP infection, Fig 2K and 2N). The mRFP fluorescence intensity of the rHEP5.0-GML-mRFP-infected neurons was significantly higher than that of the rHEP5.0-CVSG-mRFP-infected neurons in CA1 (*p* < 0.0001, one-way ANOVA; post-hoc t-test, Bonferroni-corrected, *p* < 0.00033 for rHEP5.0-CVSG-mRFP infection vs rHEP5.0-GML-mRFP infection, Fig 2F, 2L and 2O) but lower than that in CA3 (*p* < 0.0001, one-way ANOVA; post-hoc t-test, Bonferroni-corrected, *p* < 0.016 for rHEP5.0-CVSG-mRFP infection vs rHEP5.0-GML-mRFP infection, Fig 2G, 2M and 2P). Although the dendrites and axons of rHEP5.0-GML-mRFP-infected CA1 pyramidal cells can be clearly visualized, the apical dendrites showed an abnormal wavy morphology (Fig 2S). Similar to the *in vitro* results at 6 dpi (S1 Fig), the rHEP5.0-GML-mRFP-infected CA1 cells also showed a significantly higher intensity of anti-N antibody labeling than rHEP5.0-GctL-mRFP- and rHEP5.0-CVSG-mRFP-injected rats (*p* < 0.0001, one-way ANOVA; post-hoc t-test, Bonferroni-corrected, *p* < 0.00033 for rHEP5.0-CVSG-mRFP infection vs rHEP5.0-GML-mRFP infection and rHEP5.0-GctL-mRFP infection vs rHEP5.0-GML-mRFP infection, Fig 2E, 2H and 2K, and S3 Fig).

### Expression of fluorescent timer by high-transgene-expression-level RV vector

Among the propagation-competent RV vectors that we constructed, rHEP5.0-GctL was the most superior vector in terms of transgene expression and transsynaptic propagation. To further evaluate the usefulness of this vector, we constructed an RV vector that expresses a slow-fluorescent timer (sFT), which is an mCherry-derived monomeric variant that changes the color of its fluorescence from blue to red over time [18]. It has been reported that the fluorescence maxima of sFT for the blue forms is 9.8 h, and the maturation half-times for the red forms is 28 h at 37°C. Since this blue-to-red chromophore maturation speed of sFT is similar to the speed of the RV vector to cross one synapse, that is approximately 2 days [16,31], we expected that the sFT-expressing RV vector would enable us to differentiate 1^st^-order neurons from 2^nd^-order neurons in terms of the red-to-blue fluorescence intensity ratio of sFT.

**Fig 3:**
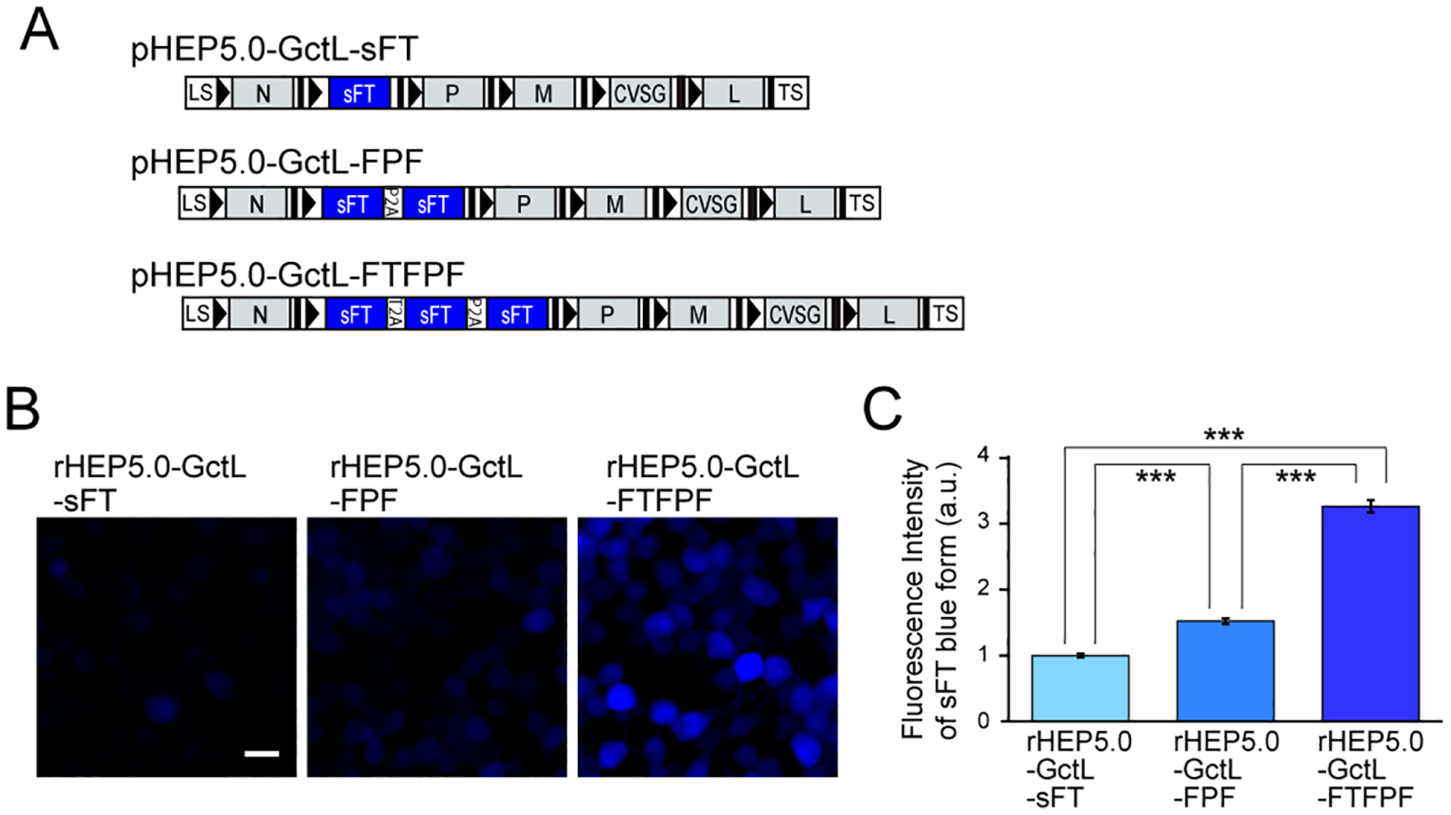
Characteristics of sFT-expressing RV vectors *in vitro*. **A**: Genome organization of recombinant RV vectors. **B-C**: Fluorescence photomicrographs of NA cells infected with sFT-expressing RV vectors (B) and fluorescence intensity of sFT in infected cells [mean ± standard errors, numbers of analyzed cells: 1199 (rHEP5.0-GctL-sFT), 924 (rHEP5.0-GctL-FPF), and 646 (rHEP5.0-GctL-FTFPF), *** p < 0.001] at 2 dpi (C). Scale bar = 20 μm.

Because of the relatively low fluorescent intensity of sFT, native sFT fluorescence was hardly detected in NA cells infected with sFT-expressing rHEP5.0-GctL (rHEP5.0- GctL-sFT, Fig 3A and 3B). To increase the expression level of sFT, we constructed an RV vector with either two- (rHEP5.0-GctL-FPF) or three-sFT genes linked by 2A sequences (rHEP5.0- GctL-FTFPF, Fig 3A). Increasing the number of inserted sFT genes markedly increased the fluorescent intensity, and rHEP5.0-GctL-FTFPF-infected cells showed an approximately twofold higher intensity of blue form fluorescence than rHEP5.0-GctL-FPF-infected cells, and a three-fold higher intensity of blue form fluorescence than rHEP5.0-GctL-sFT-infected cells at 2 dpi (*p* < 0.0001, one-way ANOVA; post-hoc t-test, Bonferroni-corrected, *p* < 0.00033 for all pairs, Fig 3C).

**Fig 4:**
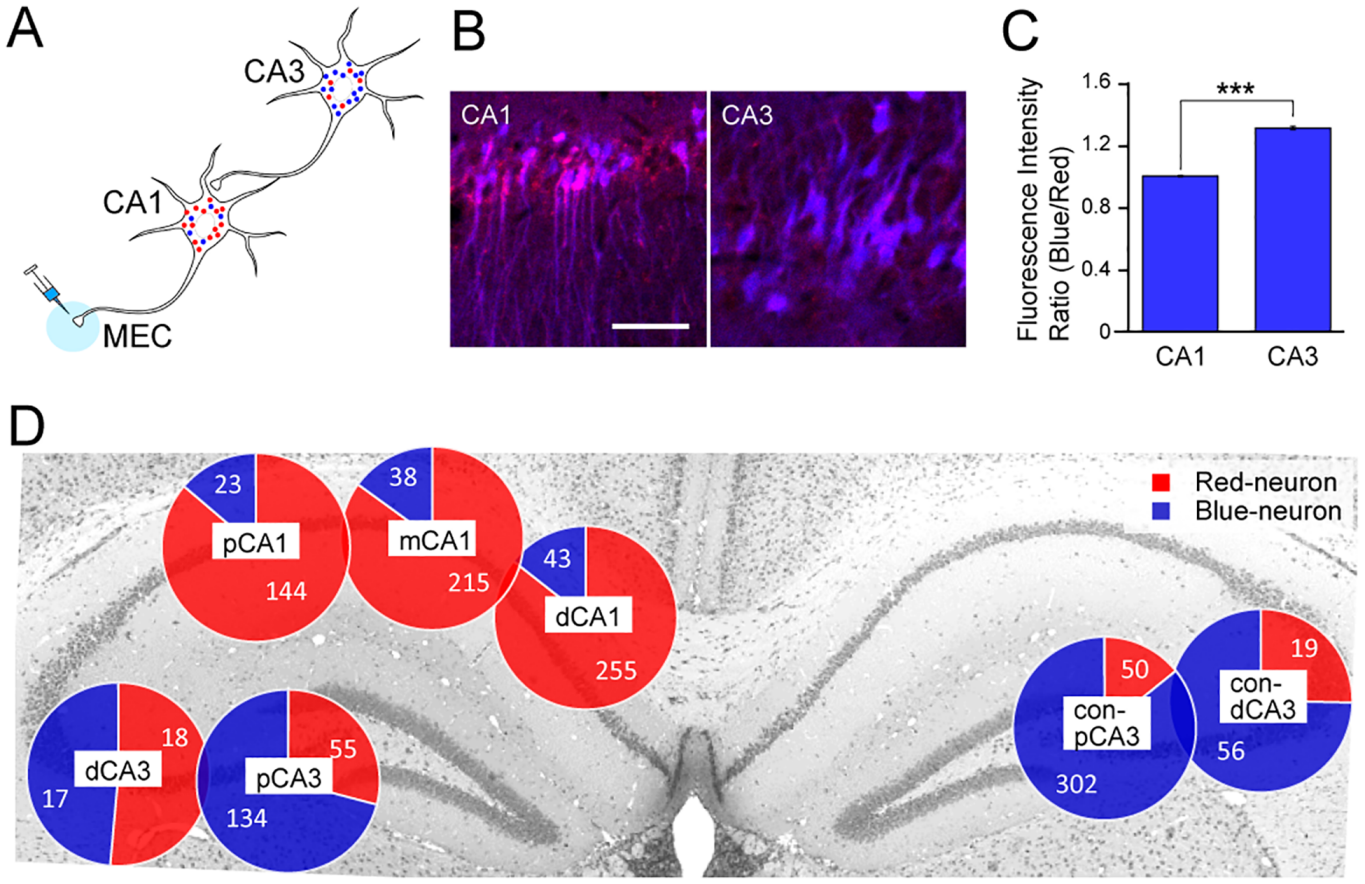
Characteristics of sFT-expressing RV vectors *in vivo*. **A**: Schematic diagram of transsynaptic tracing with sFT-expressing RV vector. **B**: Fluorescence photomicrographs of sFT fluorescence in the 1^st^-order infected area (CA1) and 2^nd^-order infected area (contralateral CA3) 6 dpi of rHEP5.0-GctL-FTFPF into MEC. Scale bar = 50 μm. **C**: Blue-to-red fluorescence intensity ratio (mean ± standard errors, numbers of analyzed cells: 717 for CA1 and 651 for CA3, *** p < 0.001) of infected CA1 and CA3 neurons. **D**: Proportions of Red-neurons and Blue-neurons in CA1 and CA3. The number in each pie chart shows the number of analyzed neurons. dCA1, distal CA1; mCA1, medial CA1; pCA1, proximal CA1; dCA3, distal CA3; pCA3, proximal CA3; con-dCA3, contralateral distal CA3; con-pCA3, contralateral proximal CA3.

We next evaluated the use of rHEP5.0-GctL-FTFPF as a transsynaptic tracer *in vivo* by injecting this vector into MEC and examining the sFT fluorescence in CA1 and CA3 (Fig 4A). After six days of survival period, sFT fluorescence was detected without any signal amplification by immunostaining in the hippocampus. Labeled neurons were observed both in the ipsilateral CA1 region and bilateral CA3 regions, which represent the 1^st^- and 2^nd^-order infected areas, respectively (Fig 4B). There were significant differences in the sFT fluorescence ratio of the blue-form to the red-form between the 1^st^- and 2^nd^-order infected area, and the ratio in CA3 was higher than that in CA1 (*p* < 0.0001, Mann-Whitney U test, Fig 4C). We also classified each sFT-labeled neuron into either the Blue- or Red-neuron based on its fluorescence ratio by k-means clustering (Fig 4D). In CA1, 85.5% of the infected neurons (614 out of 718 cells) were Red-neurons, and the proportions of Red- and Blue-neurons were similar along the transverse axis. On the other hand, 78.2% of CA3 neurons (509 out of 651 cells) were Blue-neurons, and the proportion of Blue-neurons increased in the proximal CA3 and contralateral CA3. These findings indicate that 1^st^- and 2^nd^-order neurons can be distinguished, although not perfectly, by using rHEP5.0-GctL-FTFPF.

## Discussion

In this study we aimed to develop a propagation-competent RV vector with a high transgene expression level, and have shown that rearranging the order of the M gene or shortening the IGR upstream of the L gene increase the transgene expression level. We have also shown that simply increasing the number of inserted transgenes can markedly increase the transgene expression level. These results not only enable the construction of advanced transsynaptic tracing tools but also provide useful information for further developing superior RV vectors that can be used for structural and functional analyses.

M is known to regulate the balance between viral transcription and replication, namely, inhibiting transcription while stimulating replication [24]. As we expected, replacing the order of the M and G genes resulted in an increase in transgene expression level. This can be explained by the enhancement of RV transcription, which is accompanied by the decreased expression level of M. The translocation of the M gene also resulted in reduced efficiencies of viral production and transsynaptic transport. This can also be explained by the change in M expression level since it has been reported that viral replication would be suppressed by decreasing M expression level [24]. This suppressed viral replication likely reduced viral growth rate and led to the subsequent reduction in transsynaptic transport. rHEP5.0-GML also showed increased cytotoxicity compared with rHEP5.0-CVSG. This increase in cytotoxicity can be attributed to the increased expression level of G, which is accompanied by the translocation of the G gene, since G is a major factor for cytotoxicity [9,11,32–34]. This increased cytotoxicity can also be explained by the accumulation of other viral proteins, such as the N protein, within infected cells. As shown in our *in vitro* and *in vivo* studies, the expression level of N in rHEP5.0-GML-mRFP-infected cells is higher than those in rHEP5.0-CVSG-mRFP- and rHEP5.0-GctL-mRFP-infected cells. The increase in viral transcription activity together with the suppressed viral release may have resulted in this accumulation of the N protein, which could have affected the viability of infected cells.

The transcription of the L gene, which encodes viral RNA polymerase, is attenuated by its location at the 5’ terminal and by the 24-nucleotides IGR at the G/L gene border. This attenuated expression of L results in the downregulation of virus replication and transcription, which appears to be the self-limiting strategy of RV to optimize gene expression and support prolonged host cell survival. We previously described that deleting the transgene and its transcription unit upstream of L gene enhances L gene expression. It also has been reported that replacing 24-nucleotides IGR at the G/L gene border with 2-nucleotides IGR increases L gene expression level and subsequently increases viral transcription [19]. Consistent with these findings, we succeeded in enhancing transgene expression by removing the extra transcription unit between the G and L gene and truncating the IGR upstream of L gene. Similar to this previous report, we also obtained the increase in cytotoxicity *in vitro* by shortening IGR. However, we did not see any marked difference in the morphology of infected cells *in vivo* which suggests that rHEP5.0-GctL can be used as a transsynaptic tracer. This high-transgene-expression-level vector enabled the detection of infected neurons *in vivo* without requiring any staining. No antibodies to fluorescent proteins will be required and they may be used for other purposes, such as the identification of the characteristics of infected neurons using cell-type specific molecular markers. We also showed that the transgene expression level can be further increased by simply inserting several genes linked by the 2A sequence. This will enable the use of fluorescent proteins with relatively low fluorescent intensities.

Cytotoxicity is a major issue in the field since viral vectors with low cytotoxicity would be a useful tool not only to study the connections and structures but also to monitor and manipulate the activity of targeted neurons. Although our main aim of this study was to enhance the transgene expression level, our results also provide clues to lower the cytotoxicities of G-deleted RV vectors. We previously showed that the deletion of the G gene results in a marked increase in transgene expression level and decrease in cytotoxicity [11]. This decrease in cytotoxicity is due to the lack of the G protein, which strongly affects the viability of infected cells [9,32–34]. Even with the G-deleted RV vector (rHEP5.0-ΔG), however, the basic properties of infected neurons can be maintained only for 16 days. This limitation is probably due to the high activity of viral transcription, since rHEP5.0-ΔG showed approximately twofold larger amounts of mRNAs encoding N, P, M, and L than rHEP5.0-CVSG in NA cells [21]. To further reduce the cytotoxicity of G-deleted RV vectors, we presume that it is necessary to suppress the expression of viral proteins. This may be realized by conducting the opposite manipulation, which we carried out in this study: extend the IGR in front of the L gene in order to suppress the expression of the L gene and move the position of the M gene upstream of the P gene to enhance the expression of the M gene. It may also be useful to insert an additional transcription unit with the M gene in front of the L gene. This would result in an increase in M expression level by duplicating the M gene and a decrease in the L expression level by displacing the L gene in the posterior of the genome. Although this manipulation likely affects not only viral transcription but also transgene transcription, the reduction of transgene expression can be compensated by inserting multiple transgenes linked by 2A sequences, as shown in this study. Recently, it has been reported that changing the backbone of the viral vector from the vaccine strain SAD-B19 to the fixed strain CVS-N2c markedly reduces the cytotoxicity of the vector [14]. This G-deleted RV based on CVS-N2c showed low cytotoxicity, and the infected cortical neurons maintained normal cell physiology and functional responses for up to 28 days after infection. Applying the modification described above to the CVS-N2c-based G-deleted RV vector may further improve this vector with low cytotoxicity.

Transsynaptic tracing using RV vectors is a useful method to delineate the hierarchical connections of the complicated central nervous system. However, there are two major pitfalls in this tracing method. First, samples with different survival periods must be prepared to identify the 1^st^- and 2^nd^-order neurons. The use of sFT-expressing RV vectors could be a solution to this problem, and decrease the number of animals used in tracing studies. This method, however, cannot perfectly differentiate 1^st^-order neurons from 2^nd^-order neurons, as shown in the proportion of Blue- and Red-neurons in Fig 4D. Even though it is not perfect, it is interesting that the ratio of Blue-neurons increases in proportion to the distance from the 1^st^-order neurons, such as the proximal CA3 and the contralateral CA3. Replacing sFT with a fluorescent timer, which shows chromophore maturation that perfectly matches the viral transsynaptic transport speed, could make this viral vector a superior tracing tool. The second problem is the difficulty in accurately identifying the infection pathway of the RV vector, that is, the synaptically connected circuit. The use of a G-deleted RV vector would be a solution for this problem. By supplying the G gene with other viral vectors, the G-deleted RV vector enables the identification of neurons that specifically project to targeted projection neurons [35,36]. However, it is difficult to cover the entire multisynaptic inputs to the targeted area by this method. The combination of these two RV vectors by first using the propagation-competent RV vectors, which can easily reveal overall afferent inputs to the targeted area, and then using the G-deleted RV vectors, which is useful for the accurate identification of the synaptic connections, would be a useful strategy for unraveling the complex architecture of the central nervous system.

## Supporting information

S1 Fig Characteristics of RV vectors in cultured cells after long-term survival. **A**: Photomicrographs of RV-infected NA cells at 6 dpi. The three RV vectors expressed the transgene mRFP (red), which was inserted between the N and P genes. Infection of the viral vector can be confirmed by the immunofluorescence of the N protein (green), and the number of attached cells can be observed by Hoechst staining (blue). Scale bar = 20 μm. **B-C**: Fluorescence intensities of mRFP (B) and anti-N antibody staining (C) in infected cells at 6 dpi [mean ± standard errors, numbers of analyzed cells: 76 (rHEP5.0-CVSG), 57 (rHEP5.0-GctL), 25 (rHEP5.0-GML), *** *p* < 0.001, ** *p* < 0.01]. **D**: Number of attached cells in the infected dish per that in control dish at 6 dpi (mean ± standard errors, N = 16, *** *p* < 0.0001).
(TIF)

S2 Fig Cytotoxicity of RV vectors in cultured cells. **A**: Photomicrographs of RV-infected NA cells at 2- and 5-dpi. Cells were stained with Hoechst (Blue) and subjected to TUNEL (Green). White arrow heads show TUNEL-positive cells. Scale bar = 20 μm. **B**: Percentage of TUNEL-positive cells at 2- and 5-dpi (mean ± standard errors for triplicate samples, * *p* < 0.05).
(TIF)

S3 Fig Anti-N antibody staining of RV vectors *in vivo*. **A**: Fluorescence photomicrographs demonstrating anti-N antibody staining of infected CA1 and contralateral CA3 neurons at 6 dpi after injection of either rHEP-CVSG-mRFP (E-G), rHEP-GctL-mRFP (H-J), or rHEP-GML-mRFP (K-M) into MEC. Scale bar = 1000 μm. **B-C**: Fluorescence intensity of anti-N antibody staining in CA1 (B) and CA3 (C) [mean ± standard errors, number of analyzed cells: 346 (rHEP5.0-CVSG), 225 (rHEP5.0-GctL), and 234 (rHEP5.0-GML) for CA1; 48 (rHEP5.0-CVSG), 50 (rHEP5.0-GctL), and 19 (rHEP5.0-GML) for CA3, *** *p* < 0.001].
(TIF)
