# Supplementary figures and images for: Increased transgene expression level of rabies virus vector for transsynaptic tracing
Source: PLoS One. 2017 Jul 10;12(7):e0180960. doi: 10.1371/journal.pone.0180960 (PMC5507306; doi:10.1371/journal.pone.0180960)

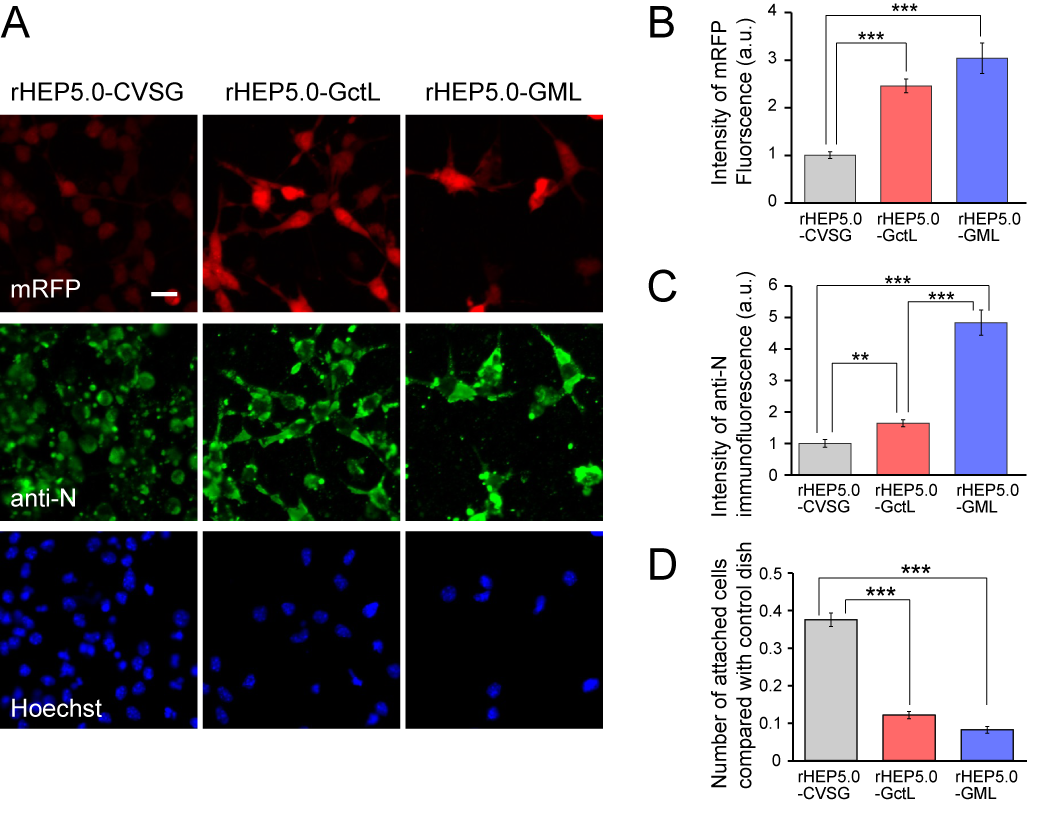

Supplement: S1 Fig — A: Photomicrographs of RV-infected NA cells at 6 dpi. The three RV vectors expressed the transgene mRFP (red), which was inserted between the N and P genes. Infection of the viral vector can be confirmed by the immunofluorescence of the N protein (green), and the number of attached cells can be observed by Hoechst staining (blue). Scale bar = 20 μm. B-C: Fluorescence intensities of mRFP (B) and anti-N antibody staining (C) in infected cells at 6 dpi [mean ± standard errors, numbers of analyzed cells: 76 (rHEP5.0-CVSG), 57 (rHEP5.0-GctL), 25 (rHEP5.0-GML), *** p < 0.001, ** p < 0.01]. D: Number of attached cells in the infected dish per that in control dish at 6 dpi (mean ± standard errors, N = 16, *** p < 0.0001). (TIF) [file pone.0180960.s001.tif]

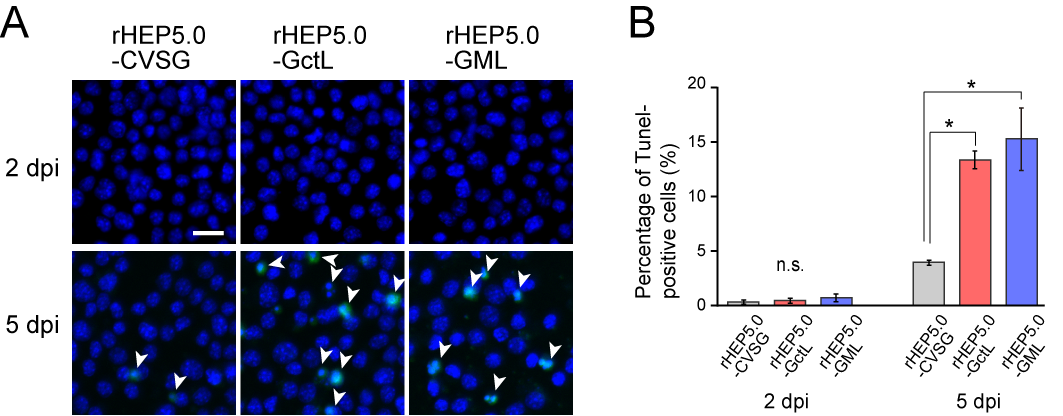

Supplement: S2 Fig — A: Photomicrographs of RV-infected NA cells at 2- and 5-dpi. Cells were stained with Hoechst (Blue) and subjected to TUNEL (Green). White arrow heads show TUNEL-positive cells. Scale bar = 20 μm. B: Percentage of TUNEL-positive cells at 2- and 5-dpi (mean ± standard errors for triplicate samples, * p < 0.05). (TIF) [file pone.0180960.s002.tif]

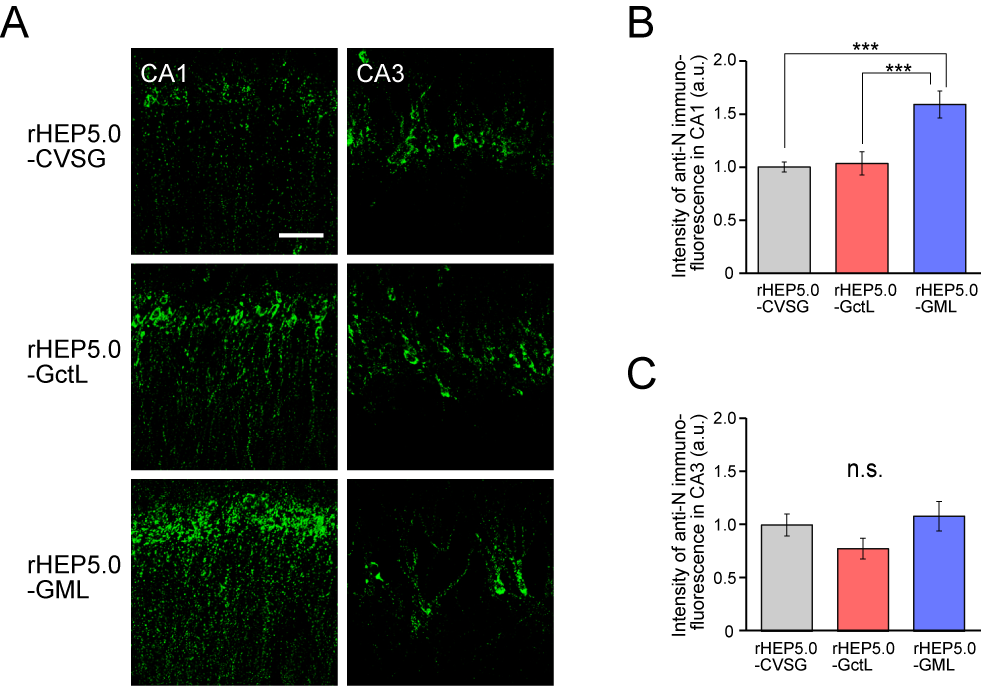

Supplement: S3 Fig — A: Fluorescence photomicrographs demonstrating anti-N antibody staining of infected CA1 and contralateral CA3 neurons at 6 dpi after injection of either rHEP-CVSG-mRFP (E-G), rHEP-GctL-mRFP (H-J), or rHEP-GML-mRFP (K-M) into MEC. Scale bar = 1000 μm. B-C: Fluorescence intensity of anti-N antibody staining in CA1 (B) and CA3 (C) [mean ± standard errors, number of analyzed cells: 346 (rHEP5.0-CVSG), 225 (rHEP5.0-GctL), and 234 (rHEP5.0-GML) for CA1; 48 (rHEP5.0-CVSG), 50 (rHEP5.0-GctL), and 19 (rHEP5.0-GML) for CA3, *** p < 0.001]. (TIF) [file pone.0180960.s003.tif]
